# Treating Traumatic Lumbosacral Spondylolisthesis Using Posterior Lumbar Interbody Fusion with three years follow up

**DOI:** 10.12669/pjms.305.5096

**Published:** 2014

**Authors:** Shujie Tang

**Affiliations:** Shujie Tang, MD, PhD, Department of Traditional Chinese Medicine, Medical school, Jinan University, Guangzhou, 510632, China.

**Keywords:** Traumatic lumbosacral spondylolisthesis, Posterior lumbar interbody fusion, Follow-up

## Abstract

***Objective:*** To analyze the surgical outcome of traumatic lumbosacral spondylolisthesis treated using posterior lumbar interbody fusion, and help spine surgeons to determine the treatment strategy.

***Methods:*** We reviewed retrospectively five cases of traumatic lumbosacral spondylolisthesis treated in our hospital from May 2005 to May 2010. There were four male and one female patient, treated surgically using posterior lumbar interbody fusion. The patients’ data including age, neurological status, operation time, blood loss, follow-up periods, X- radiographs and fusion status were collected.

***Results:*** All the cases were treated using posterior lumbar interbody fusion to realize decompression, reduction and fusion. Solid arthrodesis was found at the 12-month follow-up. No shift or breakage of the instrumentation was found, and all the patients were symptom-free at the last follow-up.

***Conclusion:*** Traumatic lumbosacral spondylolisthesis can be treated using posterior lumbar interbody fusion to realize the perfect reduction, decompression, fixation and fusion.

## INTRODUCTION

Traumatic lumbosacral spondylolisthesis is rare injury.^1^^-^^3^ and mostly published as case report. In the past, X-radiographs taken in emergency room were not so adequate that the missed diagnosis may occur in some cases, resulting in the underestimated incidence of the lesion. However, the widespread use of MRI and CT in recent decades has facilitated the early diagnosis of the injury and more cases have been reported in English literatures,^4^^-^^6^ demonstrating the frequency of the lesion may be far higher than its previous estimation.

Although some cases were treated successfully using conservative methods,^7^^,^^8^ most authors suggested the conservative treatment would result in posttraumatic translational instability or chronic low back pain and need late reconstruction.^1^^,^^3^ In addition, the lesion belongs to a three-column injury^9^ and a solid internal fixation is needed. With the improvement in medical imageology, spine surgeons learn more details about the injury. Nowadays, most authors advocate the surgical treatment for the lesion.^1^^-^^3^^,^^9^ Treatment considerations must seek to restore normal alignment, decompress the nerves and stabilize the lumbar spine, by open reduction and rigid internal fixation.^10^

However, the selection of surgical approaches remains controversial^4^ and different treatment modalities have been used for the lesion. The traumatic lumbosacral spondylolisthesis cases, reported in English literatures, were treated by a posterior approach,^11^^-^^13^ anterior approach^14^ or combined anterior and posterior approach^6^^,^^15^^,^^16^ to achieve reduction, internal fixation and fusion. However, up to now, no agreement was reached in the selection of surgical approach.

Therefore, we reviewed retrospectively the five cases of traumatic lumbosacral spondylolisthesis treated surgically in our hospital from May 2005 to May 2010, and our objectives were: 1) To analyze the characteristics and surgical outcomes of these cases, and 2) To help spine surgeons determine the treatment strategy for the lesion.

## METHODS

Between May 2005 and May 2010 five patients with traumatic lumbosacral spondylolisthesis were treated surgically in our hospital. There were four men and one woman. The average age at presentation was 39 years (range 31-46 years). A car or motorcycle accident was the cause of injury for four cases and machine crash for one case. In the five cases, there was a bilateral lumbosacral facet-dislocation in three cases, and acute spondylolytic spondylolisthesis in two cases. Before treatment, all patients complained of low back pain. In terms of the neurological status, one case had radicular symptoms, two presented with incomplete cauda equina syndrome and the other two were normal. None of the five patients had prior surgery. Patient’s data are summarised in Table-I.

X-radiographs were obtained at 6-month intervals after surgery for the first year, then yearly to assess the status of the interbody fusion. Interbody fusion was determined to be achieved if a transvertebral osseous bridge had formed anterior and posterior to the cage on the plain radiographs, if a radiolucent line between the cage and endplate was not present, if loosening or breakage of pedicle screws did not occur and if there was no motion on dynamic flexion-extension radiographs.^17^

In the current study, all the five cases were treated using posterior lumbar interbody fusion to realize the optimal reduction, decompression, fixation and fusion. Patients were placed in the prone position, and standard posterior exposure was carried out, and subtotal bilateral resection of articular processes as well as laminectomy at L5 level were performed to decompress the nerve roots and facilitate the placement of cages. Posterior pedicle screw instrumentation was placed from L5 to S1 and the reduction of the anterior slip was achieved. L5 disc was excised, and two PEEK cages were inserted posteriorly with autologous bone grafts, then posterolateral spinal fusion was performed at L5-S1 level.

## RESULTS

There were no intraoperative or postoperative complications for all cases. The average operative time was 1.8 hours (range 1.3-2.2 hours). The estimated blood loss was 300 ml (range 150–450 ml) and no patients received blood transfusion. The average length of follow-up was 44.8 months (range 36-58 months) and none was lost. Solid arthrodesis and maintenance of the reduction were found at the 12-month follow-up, and no shift or breakage of the instrumentation in all patients at the final follow-up.

In the current five patients, reduction, decompression, internal fixation and interbody fusion were performed using posterior lumbar interbody fusion, in which subtotal bilateral resection of the L5-S1 articular processes and L5 laminectomy were performed to facilitate reduction, decompression and placement of posterior interbody cages. Complete reduction of the anterior slip was achieved in all five cases. At the final follow-up, the one with radicular deficit and the two with incomplete cauda equina syndrome recovered completely, and all the patients were symptom-free.

## DISCUSSION

Traumatic lumbosacral spondylolisthesis is the result of high-energy injury, usually accompanied with multi-trauma,^7^ and the concomitant transverse process fractures were reported in most of cases.^9^ In the current study, all the cases had transverse process fractures, three of five cases had limb or rib fractures concomitantly, indicating the combination of several serious forces acted in the occurrence of the rare injury.

Most cases of traumatic lumbosacral spondylolisthesis reported in English literatures occurred in L5-S1 level, other level is very rare. In the current study, all the five cases are L5-S1 spondylolisthesis. The coronal facet orientation and lumbosacral joint angle may explain why traumatic lumbosacral spondylolisthesis occurs mostly on L5–S1 level instead of other levels.^3^ Different classifications for Lumbosacral spondylolisthesis have been published, but no classification for L4-5 or other levels published in literatures because of its rarity.

In terms of surgical approaches, Tofuku^9^, Lim^2^ and Deniz^3^ suggested the lesion should be treated using posterior approach. Grabe^14^ treated a case using anterior approach. While, Reinhold^18^ and Assuity^19^ each reported a case of traumatic lumbosacral spondylolisthesis treated using a combined anterior and posterior approach, two-stage procedure. Up to now, there is not a final criterion of approach selection for the treatment of traumatic lumbosacral spondylolisthesis.

**Table-I:** The preoperative data of the five cases

***Case No.***	***Gender***	***Age***	***Cause of Injury***	***Lumbar lesion***	***Grade of slippage***	***Associated lesions***	***Neurological status***
1	Male	36 y	Car accident	L5 spondylolisthesis, fracture of the left transverse process of L3-5	Grade-III	Multiple rib fractures	Normal
2	Male	38 y	Motorcycle accident	L5 spondylolisthesis, fracture of the left transverse process of L4.	Grade-II	Fracture of right femur	Normal
3	Male	31 y	Car accident	L5 spondylolisthesis, fracture of the right transverse process of L3-4.	Grade-I	-	Radicular pain
4	Male	41 y	Machine crash	L5 spondylolisthesis, fracture of left L1-3 transverse processes, bilateral fracture of transverse process and spinous process of L4, and fracture of spinous process of L5	Grade-II	-	Incomplete cauda equina syndrome
5	Female	46 y	Car accident	L5 spondylolisthesis, transverse process fractures of L2–3 on the left and L4 bilaterally, spinous process fractures of L2–4, and lamina fracture of L4.	Grade-II	Multiple rib fractures, fracture of left tibia	Incomplete cauda equina syndrome

**Fig.1:**
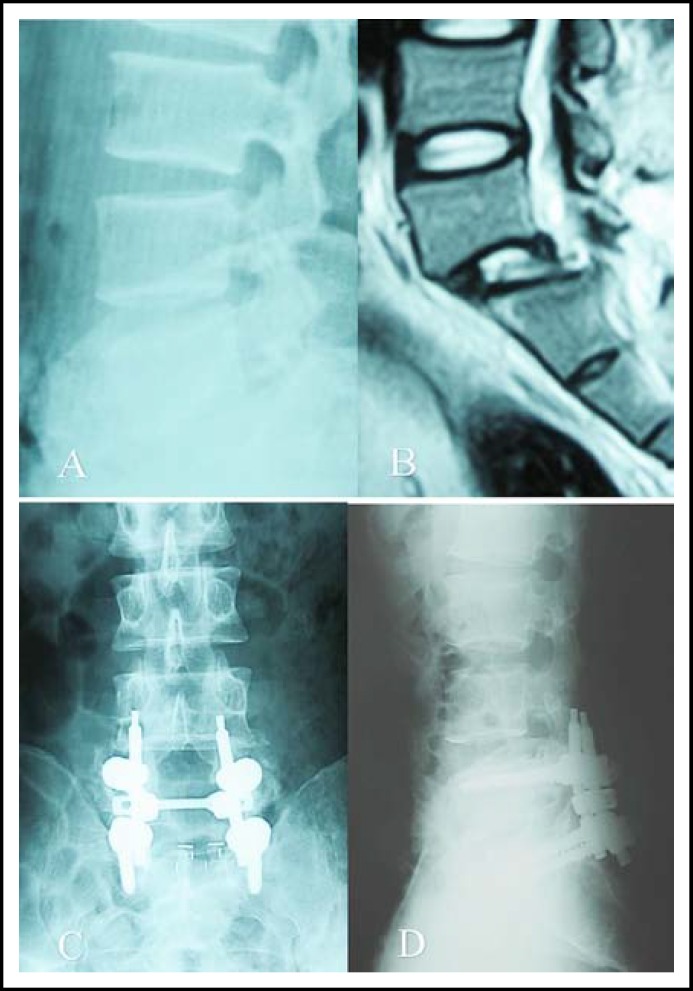
A 41-year-old male with traumatic lumbosacral spondylolisthesis, lateral X-radiographs **(A)** and MRI image **(B) **showed anterior displacement of L5 on S1, and postoperative X-radiographs **(C, D) **revealed the maintaining of reduction and solid fusion

In our opinion, a traumatic disruption of the intervertebral disc material usually occur in the injury. The neglection of the disrupted disc material may press cauda equina and aggravate the neurological symptoms,^20^ and excision of disc and interbody fusion are needed.^3^ In addition, the displacement of vertebrae need to be reduced to relieve the oppression on nerve tissues. Subsequently, the critical treatment for this injury is reduction, decompression and internal fixation to avoid further injury to the nerve system, stabilize the spine and promote the recovery of the nerve tissues. Also, in most cases, facet or laminar fracture may occur concomitantly in the injury, the fracture tips may press the nerve tissues, and need to be removed only by a posterior approach. Subsequently, compared with anterior approach, posterior approach has more advantages in treating traumatic lumbosacral spondylolisthesis. Moreover, posterior approach is of safety, easiness and minimum complication, which can avoid the occurrence of intraoperative complications resulted from anterior approach, decrease the operation time and cost.

 Some cases of traumatic lumbosacral spondylolisthesis have been treated using posterior approach, but in many of which the posterolateral fusion were performed instead of interbody fusion, resulting in breakage of the instrumentation.^2^ Interbody fusion is superior to posterolateral fusion for preventing non-union, reducing slippage and improving back pain, which is more predictable for maintaining correction and achieving union.^2^ In addition, some cases reported in the literatures were treated using two-stages, combined anterior and posterior approach, even the slippage is low-grade. In the first stage, the reduction and posterior fixation were performed, and in the second stage, an anterior interbody fusion was performed. However, we think the above procedures can be performed in posterior approach alone and similar clinical effects can be achieved. Also, some authors suggested that posterior lumbar interbody fusion has the same effect as anterior lumbar interbody fusion in fusion rate and functional outcome.^21^

In the current study, all the five patients of lumbosacral spondylolisthesis were treated surgically using posterior lumbar inter body fusion to obtain satisfactory reduction, decompression, internal fixation and inter body fusion. In addition, solid arthrodesis and maintenance of the reduction were found in all the cases, the neurological function recovered completely and all the patients were symptom-free at the final follow-up. Subsequently, we suggest that the posterior lumbar inter body fusion be the perfect method in treating traumatic lumbosacral spondylolisthesis.
